# Multi-view heterogeneous molecular network representation learning for protein–protein interaction prediction

**DOI:** 10.1186/s12859-022-04766-z

**Published:** 2022-06-16

**Authors:** Xiao-Rui Su, Lun Hu, Zhu-Hong You, Peng-Wei Hu, Bo-Wei Zhao

**Affiliations:** 1 Xinjiang Technical Institute of Physics and Chemistry, Chinese Academy of Sciences, Urumqi, 830011 China; 2 University of Chinese Academy of Sciences, Beijing, 100049 China; 3 Xinjiang Laboratory of Minority Speech and Language Information Processing, Urumqi, 830011 China; 4 School of Computer Science, Northwestern Polytechnical University, Xi’an, 710129 China

**Keywords:** Protein–protein interaction, Protein sequence, LINE, Network representation learning, Heterogeneous molecular network

## Abstract

**Background:**

Protein–protein interaction (PPI) plays an important role in regulating cells and signals. Despite the ongoing efforts of the bioassay group, continued incomplete data limits our ability to understand the molecular roots of human disease. Therefore, it is urgent to develop a computational method to predict PPIs from the perspective of molecular system.

**Methods:**

In this paper, a highly efficient computational model, MTV-PPI, is proposed for PPI prediction based on a heterogeneous molecular network by learning inter-view protein sequences and intra-view interactions between molecules simultaneously. On the one hand, the inter-view feature is extracted from the protein sequence by k-mer method. On the other hand, we use a popular embedding method LINE to encode the heterogeneous molecular network to obtain the intra-view feature. Thus, the protein representation used in MTV-PPI is constructed by the aggregation of its inter-view feature and intra-view feature. Finally, random forest is integrated to predict potential PPIs.

**Results:**

To prove the effectiveness of MTV-PPI, we conduct extensive experiments on a collected heterogeneous molecular network with the accuracy of 86.55%, sensitivity of 82.49%, precision of 89.79%, AUC of 0.9301 and AUPR of 0.9308. Further comparison experiments are performed with various protein representations and classifiers to indicate the effectiveness of MTV-PPI in predicting PPIs based on a complex network.

**Conclusion:**

The achieved experimental results illustrate that MTV-PPI is a promising tool for PPI prediction, which may provide a new perspective for the future interactions prediction researches based on heterogeneous molecular network.

## Background

Protein–protein interactions (PPIs) are essential for growth, development, differentiation and apoptosis [1]. As a result, studying PPIs is an important task and has constituted a major component of cell biochemical reaction network, which targets to reveal the functions of proteins at the molecular level. In general, the interactions between proteins are detected by some high-throughput biomedical experiments, such as yeast two-hybrid screens [2], tandem affinity purification [3] and mass spectrometric protein complex identification [4]. The results achieved by them are reliable, but they cannot response the demand of booming data growth. On the other hand, they usually suffer from time-consuming and high cost. To address above limitations, it is urgent to propose a not only low-cost, but high-efficiency computational model to identify PPIs.

With the development of computer technology, a large number of machine learning-based methods are proposed and widely applied to the field of bioinformatics in recent years [5–9]. The majority of these machine learning-based methods is feature extraction. At an early stage, the computational methods can only extract characteristics from limited information of protein, such as protein structures, phylogenetic profiles, literature knowledge, network topology and genome [10–13], and then given a pair of proteins, predict the probability of the interaction between two proteins. However, limited by the available of extra information, the methods at that time are hard to apply without pre-existing information. Thanks to the popularity of high-throughput sequencing technology, protein sequence data now has become the most available information. As a result, nowadays, the computational methods are basically constructed based on protein amino acid sequence. Moreover, most of existing works show that it is enough to predict PPIs by extracting features from protein sequence information for its well performance [9].

Sequence-based approaches typically represent the protein sequence as a vector by feature extraction methods and predict PPIs by obtained vectors [14, 15]. For example, Romero et al. [16] extract the protein sequence feature by the general-purpose numerical codification of polypeptides, which transforms pairs of amino acid sequences into a machine learning-friendly vector, whose element represents numerical descriptors of residues in proteins, then classify the unknown protein pairs with SVM. Shen et al. [17] develop another computational method to learn conjoint-triad feature from protein amino acids and achieve a high predictive accuracy of 83.90% when applied on a dataset containing 16,000 diverse PPIs. Although these protein sequence-based methods obtain promising results, there is still a room for improvement by integrating multi-source protein information. For instance, Chen et al. [18] construct a hybrid feature representation which is composed by three kinds of protein pair representations and then adopt a stacked generalization scheme that integrates five learning algorithms to predict PPIs. Wang et al. [19–21] explore the protein evolutionary feature from the prospective of the image processing techniques, which opens a new way of researching protein sequences. Though above computational methods finish the PPI prediction task well, these existing methods still discuss PPI prediction at only protein phase, ignoring the associations between proteins and any other molecules, such as miRNA, lncRNA, disease or drug. Therefore, it is feasible to predict PPIs from the view of molecular system.

To address above limitations, we propose a systematic and comprehensive model to predict PPIs by capturing inter-view protein sequences and intra-view interactions between molecules simultaneously. We firstly collect a heterogeneous molecular network with nine proven interactions across four kinds of molecules and diseases. Then, the protein inter-view feature is extracted from its sequence by k-mer method, while the intra-view feature is obtained by encoding the heterogeneous network with popular network embedding method LINE (Large-scale Information Network Embedding). Finally, the aggregation of inter-view feature and intra-view feature is sent into Random Forest (RF) to predict potential PPIs. The contributions of this work are summarized as follows:

- We develop a novel multi-view heterogeneous molecular network representation learning framework, i.e., MTV-PPI, to predict potential PPIs based on both inter-view feature and intra-view feature.
- MTV-PPI models both protein sequences and interactions between molecules to generate high representative aggregated features that are used to predict potential PPIs.
- We have conducted extensive experiments on a collected heterogeneous molecular network and the experimental results demonstrate the effectiveness of MTV-PPI.

## Materials and methods

**Fig. 1:**
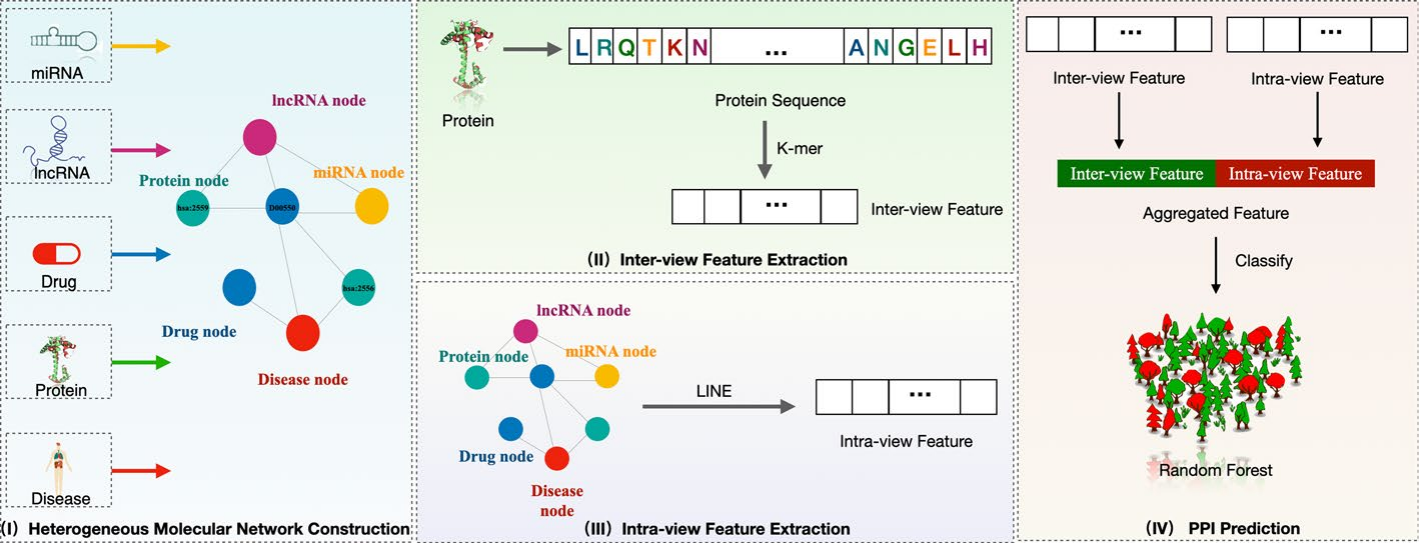
The overview of MTV-PPI

As shown in Fig. 1, MTV-PPI is composed of four steps, including i) heterogeneous molecular network construction, ii) inter-view feature extraction, iii) intra-view feature extraction, and iv) PPI prediction.

### Heterogeneous molecular network construction

To predict PPIs from a systematical perspective, we first collect existing valuable nine protein-related association datasets to construct the heterogeneous molecular network, which is shown in Table 1.

**Table 1:** The statistics of associations in the heterogeneous molecular network

Type of associations	Sources	Number
miRNA-LncRNA	lncRNASNP2 [22]	8374
miRNA-Disease	HMDD [23]	16,427
miRNA-Protein	miRTarBase [24, 25]	4944
LncRNA-Disease	LncRNADisease [26], lncRNASNP2 [22]	1264
Protein–Protein	STRING [27]	19,237
Protein-Disease	DisGeNET [28]	25,087
Drug-Protein	DrugBank [29]	11,107
Drug-Disease	CTD [30]	18,416
LncRNA-Protein	LncRNA2Target [31]	690
Total	–	105,546

As shown in Table 1, there are 19,237 validated PPIs in this collected heterogeneous molecular network, after performing the inclusion of identifier unification, de-redundancy, simplification and deletion of the irrelevant items. The statistics of constructed heterogeneous molecular network is shown in Table 2.

**Table 2:** The statistics of nodes in the heterogeneous molecular network

Type of nodes	Number
Protein	1649
LncRNA	769
miRNA	1023
Disease	2062
Drug	1025

### Inter-view feature extraction

After constructing the network, we collect the protein sequences from STRING dataset [27] for extracting inter-view feature. However, the original sequence is composed by amino acids, which is not understandable for machine. As a result, it is necessary to embed the protein sequence into a machine understandable vector before extracting protein inter-view feature. According to the polarity of the side chain, Shen et al. [17] has categorized 20 amino acids into four groups, comprising (Ala, Val, Leu, Ile, Met, Phe, Trp, Pro), (Gly, Ser, Thr, Cys, Asn, Gln, Tyr), (Arg, Lys, His) and (Asp, Glu).

**Fig. 2:**
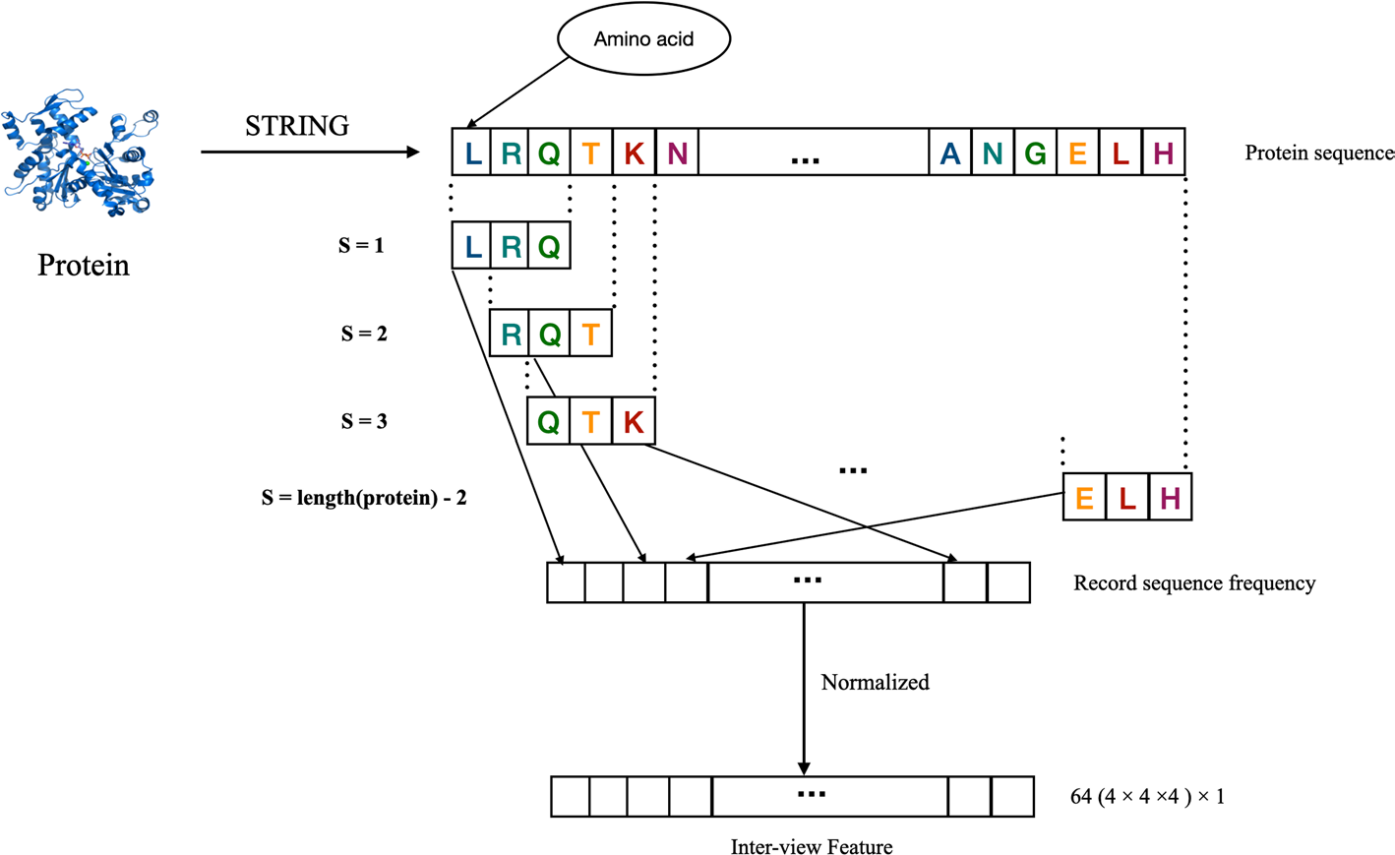
An illustration of the process of extracting inter-view feature

Inspired by Shen, we simply encode the sequences of proteins to a 64 ($4 \times 4 \times 4$) dimensional vector using the method of 3-mer. At the beginning of it, the vector is initialized to 0. Then, there is a sliding window with a length of 3, which is used to scan the whole sequence of protein with a step of 1. During that processing, the amino acid sub-sequence possessed in the window is recorded to the corresponding position of the vector. After the complement of sliding, the vector is normalized, then each dimension in the vector is the frequency at which the amino acid sequence appears in the original protein sequence. The reason for constructing 64-dimensional vectors is that there are 64 possible sorts of amino acids in four. Finally, the vector obtained by 3-mer is attribute feature. The whole process is shown in Fig. 2.

### Intra-view feature extraction

In order to predict PPIs from a global perspective, network embedding, which targets to learn the representation of nodes from an original high-dimensional space into a low-dimensional vector space, is adopted in proposed model for extracting the intra-view feature of protein from the heterogeneous molecular network. Currently, various network embedding methods are proposed and they can be generally grouped into three categories, which are Matrix Factorization (MF)-based model [32], Random Walk (RW)-based model [33, 34], and Neural Network (NN)-based model [35, 36]. Taking both efficiency and model complexity into consideration, LINE [35] is integrated into our model to learn intra-view feature of protein. LINE maps the nodes in a large network to the vector space according to the density of their relationships, so that the closely connected nodes are projected into similar locations, and the tightness of the two nodes is measured in network.

**Fig. 3:**
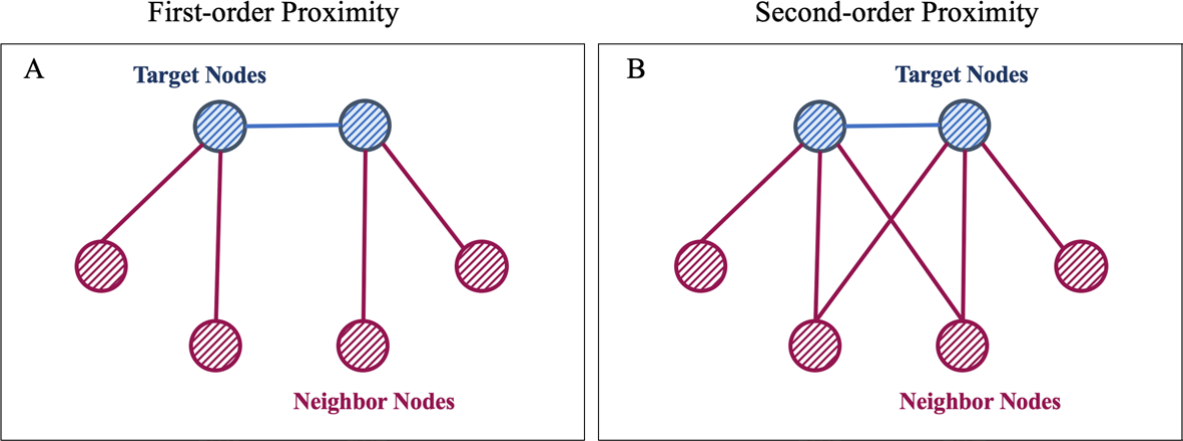
An illustration of first-order proximity and second-order proximity in LINE

For the sake of learning local and global network structures, respectively, LINE defines the first-order proximity (see Fig. 3A) and the second-order proximity (see Fig. 3B) to consider network structures at both local and global levels. The first-order proximity in the network is the self-similarity between the two nodes. For each undirected node pair $(v_i, v_j)$, the joint probability between node $v_i$ and $v_j$ can be simplied as follows:

1 $$\begin{aligned} P_{1}(v_i, v_j) = \frac{1}{1+\exp {(- \vec {v_{i}}^{T} \cdot \vec {v_{j}}})} \end{aligned}$$

where $p_{1}(v_i, v_j)$ denotes the first-order proximity between node $v_i$ and $v_j$ and $\vec {v_{i}}$ denotes the intra-view feature of node $v_{i}$.

The second-order proximity between a pair of nodes $(v_{i},v_{j})$ in a network is the similarity between their neighboring network structures. In mathematics, let $P_{v_{i}} = (p_{1}(v_{i},1), p_{1}(v_{i},2), p_{1}(v_{i},3),..., p_{1}(v_{i},|V|))$ denotes the first-order similarity between $v_{i}$ and all other nodes, then the second-order similarity between $v_{i}$ and $v_{j}$ is determined by $P_{v_{i}}$ and $P_{v_{j}}$. The second-order proximity assumes that the nodes of the shared neighbor are similar to each other. Each node plays two roles: the node itself and the neighbors of other nodes. Thus, the probability that $v_i$ is a neighbor of $v_j$ is defined as:

2 $$\begin{aligned} P_{2}(v_{i} | v_{j}) = \frac{1+\exp {(- \vec {v_{i}}^{T} \cdot \vec {v_{j}}})}{\sum _{k=1}^{|V|} \exp {(\vec {v_{k}}^{T} \cdot \vec {v_{j}})}} \end{aligned}$$

In our model, we use the above two types of proximity to optimize the intra-view features of protein nodes at the same time.

### PPI prediction

After extracting protein inter-view and intra-view features, a concatenation aggregation function is adopted to generate the final protein representation. In specific, suppose the inter-view feature and intra-view feature of node $v_{i}$ are denoted as $e_{inter}^{v_{i}}$ and $e_{intra}^{v_{i}}$, then the final representation for $v_{i}$ is formulated by:

3 $$\begin{aligned} e^{v_{i}} = \sigma (W \cdot (e_{inter}^{v_{i}}; e_{intra}^{v_{i}}) + b) \end{aligned}$$

where $e^{v_{i}}$ denotes the final representation of $v_{i}$, *W* and *b* are trainable parameters.

In this study, the PPI prediction is viewed as a binary classification task. As a result, given a protein pair, their final representations are sent into classifier to predict if the two proteins are interacted with each other and we will discuss the effect of classifier in further section.

### Performance evaluation indicators

The heterogeneous molecular network collected in this work consists of 19,237 PPIs and all of them are regarded as positive samples in MTV-PPI. To prove the effectiveness of MTV-PPI, five-fold cross-validation is adopted to train MTV-PPI. In specificity, the entire PPIs positive samples are randomly divided into five equal subsets and negative samples are randomly selected from the complement set of PPIs positive samples with an equal size for each subset. During the process of five-fold cross-validation, we take each subset as the test set and the remaining network excluding PPIs in test set as the training set, cycle five times in turn, and take the average of five times as the final performance of MTV-PPI.

Several criteria are used to evaluate proposed method, including accuracy (Acc.), sensitivity (Sen.) and precision (Pre.), Area Under Curve (AUC) and Area Under Precision-Recall (AUPR). These criteria defined below are sufficient to access the quality, robustness, and predictability of the model from different perspectives.

4 $$\begin{aligned} Acc.= & {} \frac{TN + TP}{FP + TP + FN + TN} \end{aligned}$$

5 $$\begin{aligned} Sen.= & {} \frac{TP}{TN + TP} \end{aligned}$$

6 $$\begin{aligned} Pre.= & {} \frac{TP}{TP + FP} \end{aligned}$$

where *FP*, *TP*, *FN* and *TN* represent false positive, true positive, false negative and true negative, respectively.

## Results and discussion

### Baseline algorithms

For the purpose of demonstrating the effectiveness of MTV-PPI, we compare it with several state-of-the-art baseline algorithms as follows and their performances are also evaluated in the experiments.

- **LR_PPI**^1^ [37] is a sequence-based PPI prediction model, which applies stacked auto-encoder to encode protein sequence and then predicts PPIs.
- **DPPI**^2^ [38] is also a sequence-based PPI prediction model, which applies convolutional neural network combined with random projection and data augmentation to predict PPIs.
- **WSRC_GE** [39] extracts feature from protein sequence and then introduces a novel weighted sparse representation based classifier to finish PPI prediction task.
- **LPPI**^3^ [40] reconstructs a small scale weighted network according to protein basic information, then learns the protein network representation by DeepWalk and classifies the PPI samples by Logistic Regression (LR).
- **PIPR**^4^ [41] incorporates a deep residual recurrent convolutional neural network in the Siamese architecture to predict PPIs based on protein sequences in an end-to-end way.

### Experiment settings

MTV-PPI integrates RF with default parameters to classify PPIs. For those baseline algorithms, we first download their source codes provided by their developers or ask the source codes from its developers and then apply them on the proposed heterogeneous molecular network under five-fold cross-validation on our machine. During this process, it should be noted that all the parameters used in these baseline algorithms are the same as their original works. Moreover, we randomly divide all approved PPIs as positive samples and then the same number of negative samples are randomly selected from the complement set of positive samples [42].

### Prediction performance of proposed model

In this section, we test the proposed model under five-fold cross-validation on the heterogeneous molecular network and Table 3 reports the results of each fold and the overall performance. According to the results, it can be observed that proposed model achieves the performance with 86.55% of Acc., 82.49% of Sen., 89.79% of Pre., 0.9301 of AUC value and 0.9308 of AUPR value. In addition, we also show the standard deviation of each fold and it can be seen that proposed model is stability since the average standard deviations achieved by proposed model are only 0.005 of Acc., 0.0085 of Sen., 0.0088 of Pre., 0.005 of AUC and 0.0045 of AUPR.

**Table 3:** Predictive performance under each fold on heterogeneous molecular network

Fold	Acc.	Sen.	Pre.	AUC	AUPR
0	0.8703	0.8264	0.9060	0.9341	0.9346
1	0.8732	0.8332	0.9056	0.9370	0.9378
2	0.8602	0.8181	0.8933	0.9234	0.9268
3	0.8617	0.8342	0.8828	0.9298	0.9270
4	0.8620	0.9124	0.9019	0.9262	0.9277
Overall	**0.8655** ± **0.0050**	**0.8249** ± **0.0085**	**0.8979** ± **0.0088**	**0.9301** ± **0.0050**	**0.9308** ± **0.0045**

Best results are bolded

### Comparison with baseline models

**Fig. 4:**
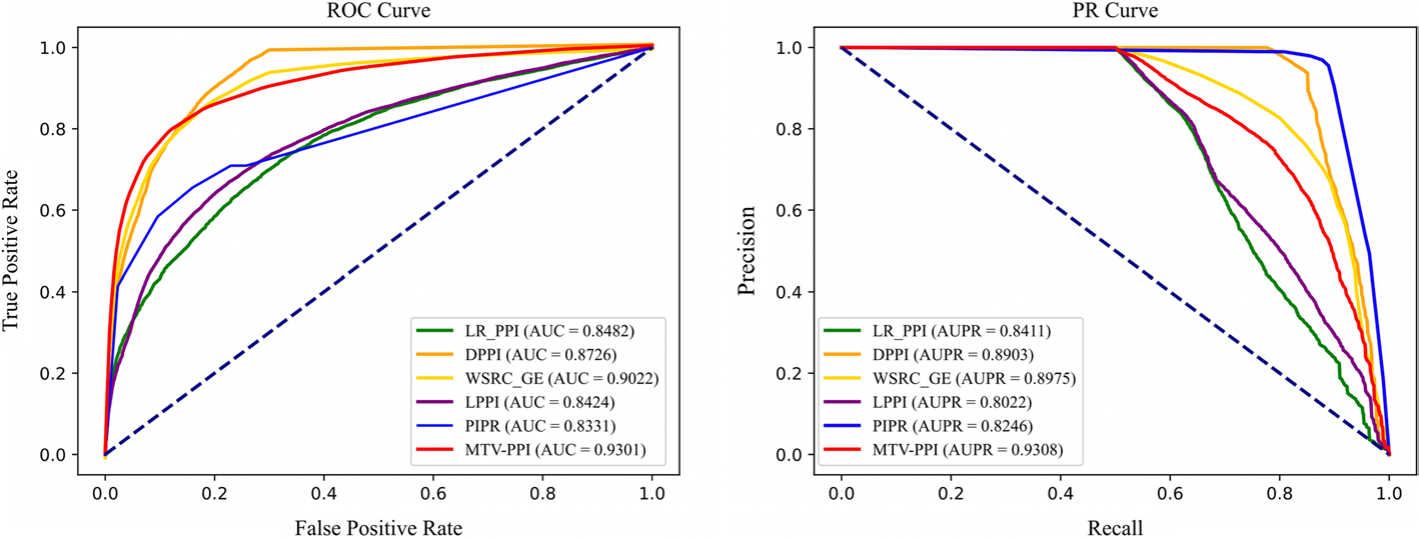
ROC and PR curves obtained by MTV-PPI and all baseline algorithms

We reimplement all baseline models on our machine and the results are shown in Table 4 and Fig. 4. Regarding the results obtained by MTV-PPI and all baseline algorithms, we find that the performances of these algorithms vary greatly and proposed method MTV-PPI achieves better results on most metrics. Compared with sequence-based algorithms (LR$\_$PPI, DPPI, PIPR and WSRC$\_$GE), MTV-PPI yields the best performance, improving the performance by approximately 7% on Acc., 5% on Sen., 15% on Pre., 0.08 on AUC and 0.08 on AUPR. The good performance is due to that MTV-PPI is capable of learning complex feature from the heterogeneous network and aggregating it with sequence-based feature. Moreover, though LPPI predicts PPIs based on network, its performance is not as good as MTV-PPI by and large. However, it achieves better result on Sen. with about 10% higher when compared with MTV-PPI and this result is also better than that of all baseline algorithms. The possible reasons for this are two folds: (i) LPPI only uses protein properties to reduce the scale of network, but these properties are not adopted to the further process of LPPI, while MTV-PPI integrates protein attribute feature into final feature, which enrich the feature to a certain extent; (ii) LPPI may lose information in the process of reducing the size of network when applied on the heterogeneous molecular network, while MTV-PPI is able to mine high-dimensional feature through on the whole heterogeneous molecular network.

**Table 4:** Results of various methods

Methods	Acc.	Sen.	Pre.	AUC	AUPR
LR$\_$PPI	0.7717 ± 0.0066	0.7551 ± 0.0090	0.7329 ± 0.0092	0.8482 ± 0.0060	0.8411 ± 0.0058
DPPI	0.8007 ± 0.0087	0.7623 ± 0.0099	0.7677 ± 0.0090	0.8726 ± 0.0076	0.8903 ± 0.0078
WSRC$\_$GE	0.8225 ± 0.0105	0.7623 ± 0.0097	0.7987 ± 0.0123	0.9022 ± 0.0089	0.8975 ± 0.0086
LPPI	0.8062 ± 0.0116	**0.9275** ± **0.0124**	0.7232 ± 0.0103	0.8424 ± 0.0173	0.8022 ± 0.0154
PIPR	0.7536 ± 0.0090	0.7678 ± 0.0100	0.7456 ± 0.0098	0.8331 ± 0.0094	0.8246 ± 0.0096
MTV-PPI	**0.8655** ± **0.0050**	0.8249 ± 0.0085	**0.8979** ± **0.0088**	**0.9301** ± **0.0050**	**0.9308** ± **0.0045**

Best results are bolded

### Impact of aggregation function

**Fig. 5:**
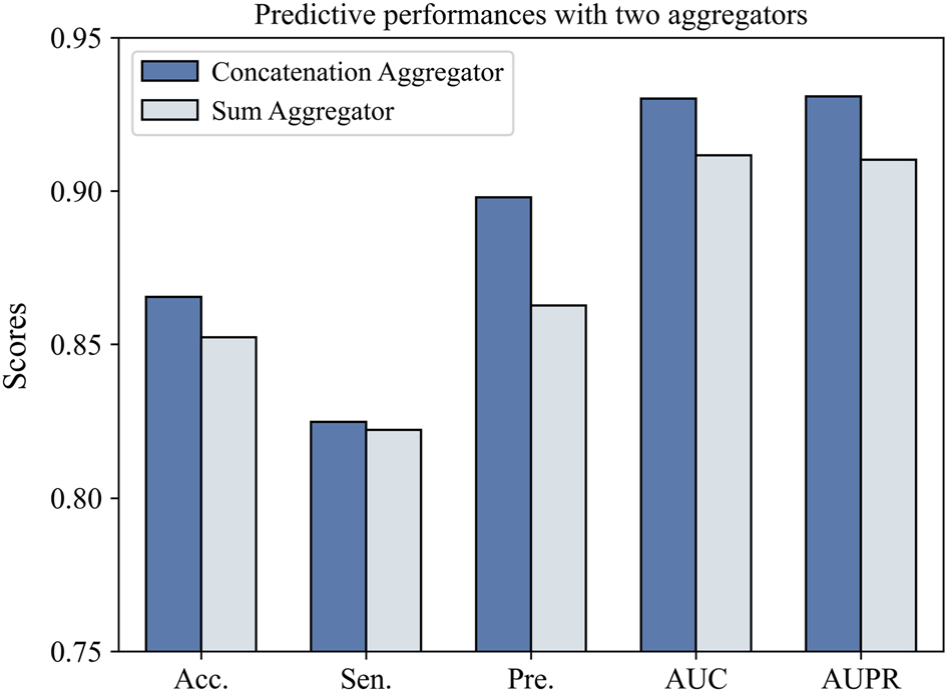
Predictive performances with two different aggregators

The inter-view feature and intra-view feature are aggregated in a concatenation way. In order to prove the effectiveness of adopted aggregation function, we compare it with another widely used sum aggregation function [43], which is formulated by: $e^{v_{i}} = \sigma (W \cdot (e_{inter}^{v_{i}} + e_{intra}^{v_{i}}) + b)$, where *W* and *b* are trainable weights. Figure 5 reports the results obtained by above two aggregators. It should be noted that the other parts of this variant are all the same as MTV-PPI except the aggregator.

According to the results shown in Fig. 5, we have found that concatenation aggregator adopted in MTV-PPI is superior to sum aggregator. The possible reason is that sum aggregator tends to detect the potential interaction between two features [43], which may not suitable for our model since the features used in MTV-PPI are extracted from two separate views.

### Impact of network representation learning algorithm

**Fig. 6:**
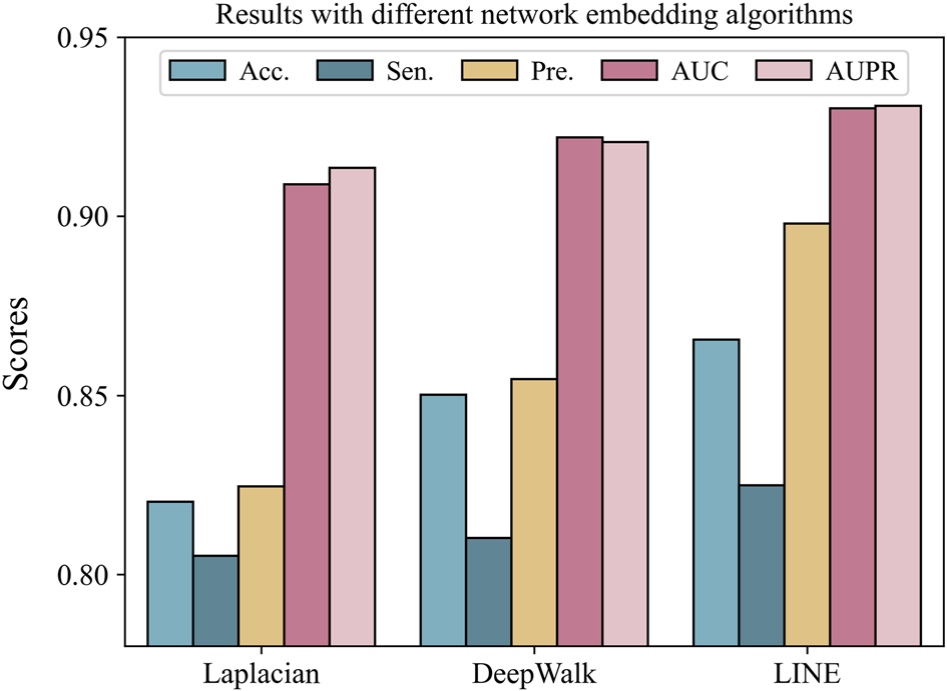
Results with different network embedding algorithms

In MTV-PPI, the intra-view feature is learned by a kind of NN-based representation learning methods, LINE. In this section, we also implement Laplacian and DeepWalk that belong to MF-based group and RW-based group, respectively, to validate the usefulness of LINE in current task. Figure 6 summarizes the experimental results and it can be observed that neither Laplacian nor DeepWalk is as effective as LINE, which may mainly because that both of them do not directly model the network topology, since Laplacian learns the low-dimensional representations of protein nodes by MF and DeepWalk learns representations through network paths, while LINE designs two kinds of topological similarities to learn low-dimensional representations for protein nodes.

### Impact of various feature representation

**Fig. 7:**
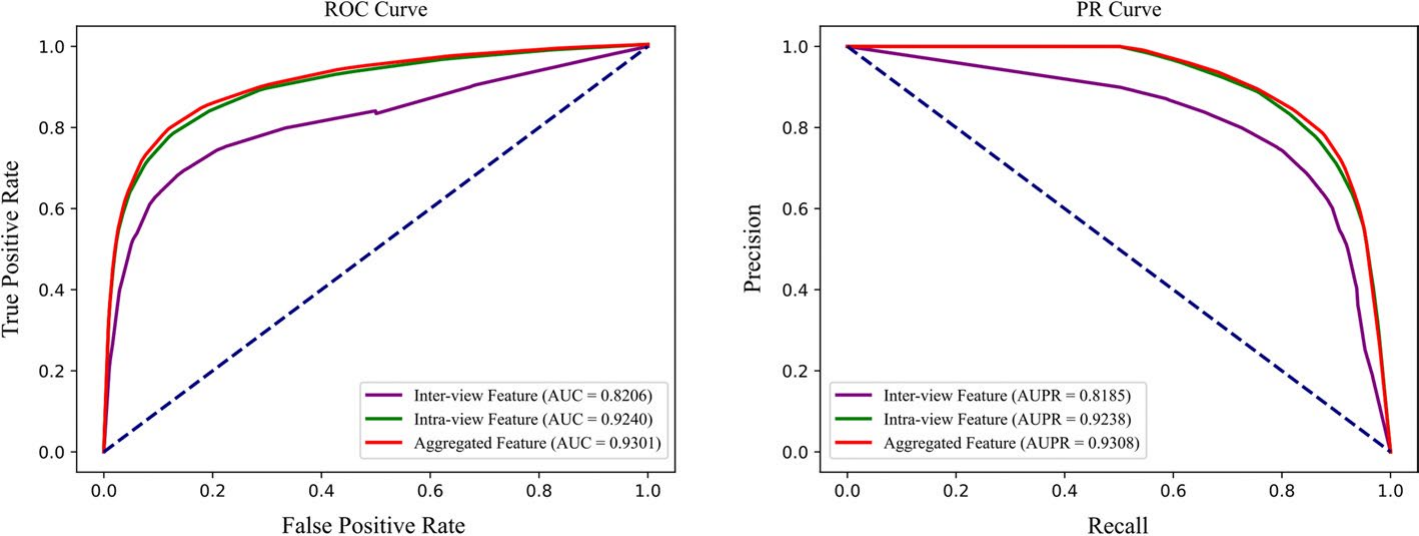
ROC and PR curves obtained by various features

As mentioned above, MTV-PPI is capable of modeling both inter-view feature and intra-view feature simultaneously. In this section, we design two variants to detect the effects of above two features, respectively. The first one only takes inter-view feature into account, while the second one predicts PPIs only by intra-view feature. We also train and test them under five-fold cross-validation. Table 5 reports their performances and Fig. 7 shows their ROC and PR curves, respectively.

**Table 5:** Predictive performance with different feature type

Feature Type	Acc.	Sen.	Pre.	AUC	AUPR
Inter-view feature	0.7491 ± 0.0090	0.6945 ± 0.0109	0.7797 ± 0.0103	0.8206 ± 0.0080	0.8185 ± 0.0181
Intra-view feature	0.8570 ± 0.0045	0.8130 ± 0.0105	0.8916 ± 0.0099	0.9240 ± 0.0046	0.9238 ± 0.0093
Aggregated feature	**0.8655** ± **0.0050**	**0.8249** ± **0.0085**	**0.8979** ± **0.0088**	**0.9301** ± **0.0050**	**0.9308** ± **0.0045**

Best results are bolded

According to the results, it can be observed that the model with only inter-view feature gets the worst performance among all metrics, which indicates that it is insufficient to predict PPIs on the heterogeneous molecular network with only feature extracted from protein sequence. Compared with inter-view feature, the model with intra-view improves the performance by 10.79% on Acc., 11.85% on Sen., 11.19% on Pre., 0.1034 on AUC and 0.1053 on AUPR, which demonstrates that intra-view feature is more conductive to PPI prediction task on heterogeneous molecular network. Though intra-view feature performs much better than inter-view feature, the model with aggregated feature achieves the best performance. The reason for this is that aggregated feature contains both two features and is able to fuse two features in an appropriate proportion.

### Impact of various machine learning classifiers

**Fig. 8:**
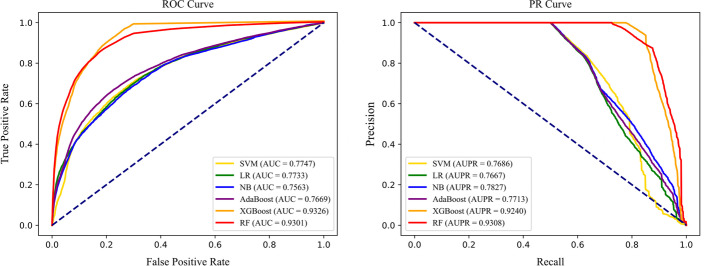
ROC and PR curves obtained by various classifiers

In proposed model, RF classifier is integrated as the default classifier. For the sake of proving the effectiveness of it, we select several state-of-the-art machine learning classifiers, including SVM [44], LR [45], Naïve Bayes (NB) [46], AdaBoost [47] and XGBoost [48], and apply them on the same heterogeneous molecular network with aggregated feature. All the other parameters are the same as original work. Table 6 and Fig. 8 shows the results of each classifier.

**Table 6:** Predictive performance with various classifiers

Classifier	Acc.	Sen.	Pre.	AUC	AUPR
SVM	0.7103 ± 0.0078	0.7577 ± 0.0113	0.6921 ± 0.0074	0.7747 ± 0.0077	0.7686 ± 0.0074
LR	0.7056 ± 0.0072	0.7452 ± 0.0119	0.6905 ± 0.0067	0.7733 ± 0.0078	0.7667 ± 0.0076
NB	0.6772 ± 0.0084	0.7392 ± 0.0098	0.6578 ± 0.0090	0.7563 ± 0.0071	0.7827 ± 0.0075
AdaBoost	0.6946 ± 0.0088	0.7306 ± 0.0115	0.6816 ± 0.0090	0.7669 ± 0.0094	0.7713 ± 0.0086
XGBoost	0.8600 ± 0.0081	**0.8867** ± **0.0063**	0.8419 ± 0.0109	**0.9326** ± **0.0051**	0.9240 ± 0.0048
RF	**0.8655** ± **0.0050**	0.8249 ± 0.0085	**0.8979** ± **0.0088**	0.9301 ± 0.0050	**0.9308** ± **0.0045**

Best results are bolded

According to the results, the two linear classifiers (SVM and LR) have the similar performances in predicting PPIs, but it yields about 16% lower than default classifier (RF) among all metrics on average, which indicates that linear classifier is not suitable to process the feature extracted from such a complex network. As for the generation model, i.e. NB, it gets the worst performance with approximately 20% lower on Acc. than that of RF classifier. The possible reason for this is that NB classifier is constructed based on the assumption that each feature of the sample is independent [49], which is not suitable for proposed task. Though AdaBoost, XGBoost and RF all belong to integrated model, their performances are quite different. Among three classifiers, AdaBoost performs worst on classifying PPI samples, while XGBoost improves the performance by about 17% on Acc., 15% on Sen., 16% on Pre., 0.17 on AUC and 0.15 on AUPR. The possible reason is that XGBoost introduces regulations and the pruning strategy to better fit the positive samples, which is also the reason why XGBoost achieves high Sen. and AUC. However, RF achieves the best results on all metrics and it is more stable than others since it has smaller standard deviations. As a result, we finally select RF as default classifier of our model.

### Impact of the type of heterogeneous molecular network

We have proved that heterogeneous molecular network helps to improve the performance of PPIs predictor in the above section. However, there are five types of nodes in the network used in this paper, including miRNA, lncRNA, Drug, Disease, and Protein, which makes it difficult to determine which types of nodes/edges benefit to PPI prediction. To this end, we construct five sub-networks as shown in Table 7 and apply MTV-PPI on them under five-fold cross-validation to determine which type of network is the most informative. Table 8 reports the experimental results obtained on each sub-network and it can be observed that: (i) Among five sub-networks, DrPP contributes the most to PPIs prediction as its superior performance when compared MiPP, LncPP and DiPP; (ii) Integrating miRNA into protein–protein network also significantly improves the performance of MTV-PPI; (iii) As for LncPP and DiPP, the effect of them is not obvious, even if the results on them are better than that of PP. In a word, DrPP is the most informative network for PPI prediction.

**Table 7:** The detail information of each subnetwork

Name	# Nodes	# Interactions
Protein–protein (PP)	1649	19,237
miRNA–protein–protein (MiPP)	2672	24,181
lncRNA–protein–protein (LncPP)	2418	19,927
Disease–protein–protein (DiPP)	3711	44,324
Drug–protein–protein (DrPP)	2674	30,344

**Table 8:** Experimental results obtained on each sub-network

Sub-network	Acc.	Sen.	Pre.	AUC	AUPR
PP	0.8348 ± 0.0065	0.7869 ± 0.0094	0.8704 ± 0.0075	0.9047 ± 0.0038	0.9144 ± 0.0041
MiPP	0.8420 ± 0.0022	0.7936 ± 0.0055	0.8786 ± 0.0049	0.9095 ± 0.0020	0.9198 ± 0.0022
LncPP	0.8350 ± 0.0044	0.7865 ± 0.0086	0.8711 ± 0.0081	0.9042 ± 0.0028	0.9143 ± 0.0028
DiPP	0.8352 ± 0.0048	0.7796 ± 0.0064	0.8772 ± 0.0068	0.9053 ± 0.0031	0.9139 ± 0.0032
DrPP	0.8537 ± 0.0034	0.8057 ± 0.0052	0.8913 ± 0.0026	0.9213 ± 0.0039	0.9291 ± 0.0040
All	**0.8655** ± **0.0050**	**0.8249** ± **0.0085**	**0.8979** ± **0.0088**	**0.9301** ± **0.0050**	**0.9308** ± **0.0045**

Best results are bolded

## Conclusion

In this paper, we propose a computational model MTV-PPI to predict PPIs through a heterogeneous molecular network by modeling both inter-view feature and intra-view feature simultaneously. The inter-view feature is used to characterize the information of protein sequence, while intra-view feature is used to describe the network structure. MTV-PPI aggregates both two features and predict potential PPIs by RF classifier. By this way, MTV-PPI is capable of taking both protein sequence information and network structure into account. Obtained experiment results show that the aggregated feature contributes to the improvement of model performance and further experiment results indicate that MTV-PPI is a promising tool for predicting PPIs based on the heterogeneous molecular network. In further work, we are going to expand the scale of the network by adding more molecules [50], incorporate the relation semantics [51], and clustering technology [52, 53] to reduce the noises in heterogeneous network into our feature work.

## Data Availability

The datasets generated and analyzed during the current study are available in https://github.com/Blair1213/MTV-PPI.

## Abbreviations

PPI: Protein–Protein interaction
SVM: Support vector machine
LINE: Large-scale information network embedding
RF: Random forest
MF: Matrix factorization
RW: Random walk
NN: Neural network
Acc.: Accuracy
Sen.: Sensitivity
Pre.: Precision
AUC: Area under curve
AUPR: Area under precision-recall
FP: False positive
TP: True positive
FN: False negative
TN: True negative
LR: Logistic regression
NB: Naive Bayes
MTV-PPI: Multi-view protein–protein interaction

## Footnotes

1 https://github.com/rasmusbergpalm/DeepLearnToolbox.

2 https://github.com/hashemifar/DPPI/.

3 https://github.com/Blair1213/LPPI.

4 https://github.com/muhao chen/seq_ppi.git.
